# Hypercalcemia-induced acute kidney injury in a Caucasian female due to radiographically silent systemic sarcoidosis 

**DOI:** 10.5414/CNCS109513

**Published:** 2018-09-04

**Authors:** Ramy M. Hanna, Marian Kaldas, Farid Arman, Melissa Wang, Terrance Hammer, Deren Sinkowitz, Anjay Rastogi

**Affiliations:** 1 Division of Nephrology,; 2 Division of Rheumatology, Department of Medicine,; 3 Department of Family Medicine, and; 4 Division of Pulmonary and Critical Care, Department of Medicine, David Geffen School of Medicine, University of California Los Angeles (UCLA), Los Angeles, CA, USA

**Keywords:** hypercalcemia, acute kidney injury, sarcoidosis, granuloma, hypervitaminosis D

## Abstract

Abstract. Sarcoidosis is a rare autoimmune disease resulting in formation of non-necrotizing “non-caseating” granulomas generally in the lung. The disease classically strikes African American females in their fourth and fifth decades. The resulting hypercalcemia is a result of 1-α hydroxylase overexpression in granulomas with increased 1,25-dihydroxy vitamin D levels. This phenomenon can also be observed in mycobacterial and fungal infections that produce granulomas in infected patients. Thus, chronic infectious diseases are part of differential diagnosis of granulomatous processes. We present an elderly Caucasian female who presented with hypercalcemia with serum calcium of 11 – 14 mg/dL and an elevated ionized calcium of 1.4 – 1.5 mmol/L. Initially cholecalciferol supplements were stopped, but hypercalcemia persisted for more than 2 months. 1,25-dihydroxy vitamin D levels were markedly elevated with low normal 25-hydroxy vitamin D levels, angiotensin-converting enzyme levels were also high, and chest computed tomography (CT) imaging was negative for any lymphadenopathy (including perihilar lymphadenopathy). Malignancy and infectious workups were negative for fungal and mycobacterial infections. Positron emission tomography revealed several small lymph nodes in right upper lobe of lung, and biopsy of bone marrow and lung lymph-nodes revealed non-caseating granulomata. We present an atypical case of occult sarcoidosis presenting mainly with biochemical findings without any definitive imaging findings, making diagnosis a clinical challenge.

## Introduction 

Sarcoidosis is a rare disease that occurs due to formation of granulomas in affected patients. These granulomas most often occur in the lungs and sometimes in the liver. They produce 1-α hydroxylase and angiotensin-converting enzyme. The 1-α hydroxylase results in the classical finding of hypercalcemia, and the angiotensin-converting enzyme is sometimes used as a marker associated with the diagnosis of sarcoidosis [1]. Sarcoidosis is most commonly found in African American women in the United States, and it is this population that is afflicted with the most severe disease and the highest rate of progression [2]. Though this is not often well appreciated, sarcoidosis occurs in all racial groups and is actually most common worldwide in patients of northern European ancestry [1]. 

The classical finding of sarcoidosis is bilateral hilar and perihilar lymphadenopathy, hypercalcemia, and lung findings [3]. Indeed sarcoidosis may be the initial presentation of lung disease that develops into interstitial lung disease. Lymphoma, malignancy, tuberculosis, and fungal infections are all on the classical differential diagnosis in patients with hilar lymphadenopathy [4]. Generally, the diagnosis is made with lymph node biopsy demonstrating non-caseating granulomata (non-necrotizing) in the correct clinical context [3, 4]. Sarcoidosis has variable imaging findings since it is usually divided into stages depending on radiographic appearance; this classification is the Siltzbach classification. Stage 0 occurring in up to 10% of patients shows no abnormalities, stage 2 shows lymphadenopathy in 50% of patients, stage 3 shows lymphadenopathy and pulmonary infiltration in 25 – 30% of patients, stage 3 shows increased pulmonary infiltration in up to 10% of patients, and in 5% of patients the end-stage presentation of fibrosis is found [5]. Sarcoidosis can also be found in various other target organs including neuro-sarcoidosis, cardiac sarcoidosis, and renal disease due to sarcoidosis [6, 7]. 

Given the variety of stages at which sarcoidosis is discovered and the multisystem nature of the disease, it is common to have atypical diagnoses [8, 9]. There have been cases of cardiac sarcoidosis that were completely silent with exception of sudden cardiac arrhythmias [10], some silent cases result in lung dysfunction that is only diagnosed on post-mortem examinations [11]. We present a patient who presented solely with hypercalcemia, elevated 1,25-dihydroxy vitamin D, and a high angiotensin-converting enzyme level. Despite the suggestive biochemical profile, the patient’s high-resolution CT showed no evidence of lung disease. Diagnosis eventually was made on biopsy allowing treatment of hypercalcemia that had resulted in progressive renal injury. 

## Case report 

We report a 77-year-old Caucasian female who had presented to nephrology clinic with a history of multiple sclerosis in remission who was noted to have progressive weakness for 2 months prior to presentation. Suspicion for a multiple sclerosis flare was low since her electrolytes were grossly abnormal with a serum calcium of 13.7 mg/dL and a serum creatinine of 2 mg/dL on June 23, 2017, increased from baseline of 8 – 9 mg/dL (calcium) and 0.9 – 1.1 mg/dL (creatinine). Ionized calcium was measured at 1.54 mmol/L (reference range 1.09 – 1.29 mmol/L), confirming the hypercalcemia noted on chemistry. Patient had initially been taking cholecalciferol 2,000 units PO daily for osteoporosis prophylaxis which were stopped, but this failed to improve the serum calcium. Parathyroid hormone (PTH) was appropriately suppressed at low normal 18 – 21 pg/mL (reference range 11 – 51 pg/mL), PTH-related peptide was in range at 17 pg/mL (reference range 14 – 27 pg/mL) which was not consistent with hypercalcemia of malignancy. The patient had normal sodium and alkaline phosphatase values. Urinalysis showed only 1+ proteinuria and was otherwise normal, and the kidney ultrasound demonstrated normal kidney structure. 25-hydroxy vitamin D level was 28 – 37 ng/mL (reference range: 20 – 50 ng/mL), but 1,25-dihydroxy vitamin D levels remained elevated despite stopping any supplements and remained elevated for nearly 2 months. 1,25-dihydroxy vitamin D peak level was 158 pg/mL (reference range: 19.9 – 79.3 pg/mL) and remained elevated between 100 and 113 pg/mL despite stopping vitamin D supplements. Urine protein electrophoresis and serum electrophoresis were negative or an M-spike, immunofixation was only positive in serum with IgG-κ monoclonal being found. κ- and λ-light chains were only slightly skewed towards IgG-κ > IgG-λ with a ratio of 2.32, ruling out monoclonal gammopathy as the etiology for the hypercalcemia. 

**Figure 1.**
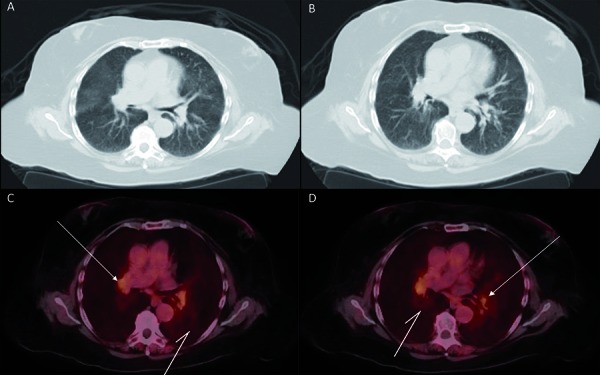
A, B: High-resolution computed tomography (perihilar slice) showing normal lung parenchyma and without any lymphadenopathy; C, D: Positron emission tomography scan with flourodeoxyglucose (FDG) showing right upper lung lobe lymphadenopathy (white two-pronged arrows) and increased FDG uptake of lung parenchyma (white one-pronged arrows).

The patient’s hypercalcemia continued to cause acute kidney injury, her calcium reached 12.6 mg/dL, she was hospitalized where she was given IV fluids, furosemide, and pamidronate. A high resolution CT scan did not show any lymphadenopathy or pulmonary pathology (Figure 1), and angiotensin-converting enzyme levels were elevated at 100 U/L and 102 U/L for 2 draws. After the aforementioned treatment, her serum calcium decreased to 10.5 mg/dL. 

When her serum calcium increased to 11.8 mg/dL, she was given denosumab 120 mg formulation in the interim leading to the return of serum calcium to 10.5 mg/dL. Since infectious causes of granulomatous diseases, such as tuberculosis and fungal infections, were in the differential diagnosis corticosteroids were not given empirically. While the high level of angiotensin-converting enzyme suggested possible sarcoidosis, we considered the low specificity of the test to demand more definitive tissue diagnosis could be obtained. Thus, corticosteroids were deferred until definite diagnosis, and denosumab was prescribed in the interim to control hypercalcemia. 

**Figure 2.**
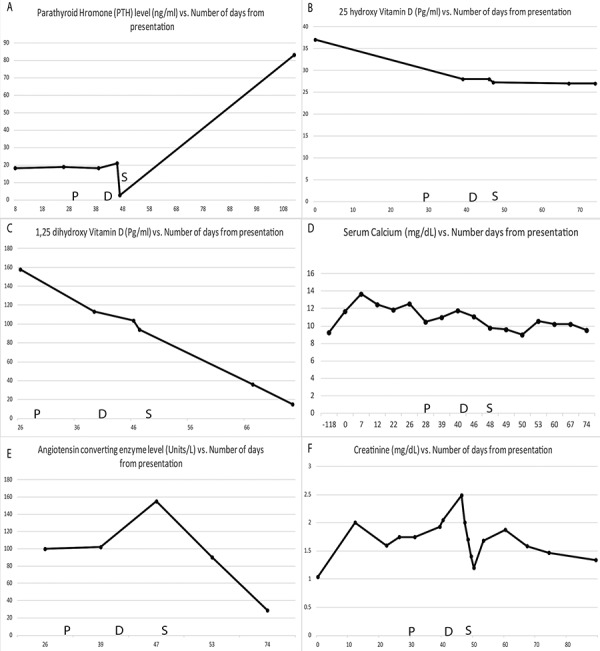
A: Parathyroid hormone (pg/mL) vs. number of days after presentation (June 15, 2017); B: Vitamin D2 (pg/mL) vs. number of days after presentation; C: Vitamin D3 (pg/mL) vs. number of days after presentation; D: Serum calcium (mg/dL) vs. number of days after presentation; E: Angiotensin-converting enzyme (U/mL) vs. number of days after presentation; F: Serum creatinine (mg/dL) vs. number of days after presentation. P = pamidronate injection (29 days after presentation); D = denosumab injection (41 days after presentation); S = start of prednisone (48 days after presentation).

See Figure 2 for graphs of serum calcium, PTH, 25-hydroxy vitamin D, 1,25-dihydroxy vitamin D, angiotensin-converting enzyme level, and serum creatinine levels. Mycobacterial serologies, coccidiomycosis, and other fungal serologies were negative as well. 

It was during this time that granulomatous disease, specifically sarcoidosis was suspected. She underwent PET scan with findings of upper lobe predominant peri-bronchial wall thickening, diffuse lymphadenopathy and flourodeoxyglucose (FDG) uptake throughout pulmonary parenchyma. This was suggestive of a granulomatous process such as sarcoidosis. No other focus of FDG uptake in a pattern that suggests malignancy was found, and no splenic involvement was seen. See Figure 1 for PET scan image and findings. 

**Figure 3.**
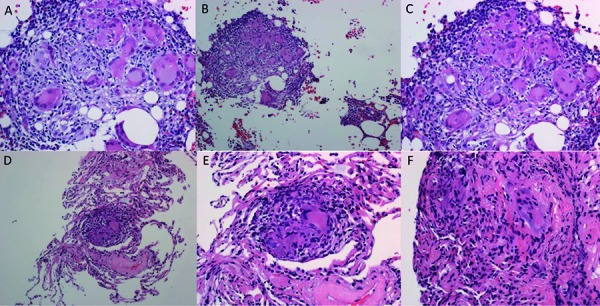
A, B, C: Lung biopsy findings showing non-caseating granulomas; D, E, F: Bone marrow biopsy findings showing non-caseating granulomas. Stains: Hematoxylin and eosin stains; Magnification: high power 400× (A, B, C) and low power 100× (D, E, F).

When the patient’s creatinine increased to 2.58 mg/dL with a serum calcium of 11 mg/dL, the patient was admitted for definitive diagnostic procedures. Bone marrow biopsy and a transbronchial lymph node biopsy were obtained. The patient’s serum calcium and serum creatinine normalized with hydration and subcutaneous calcitonin injection. Biopsy results showed diffuse non-necrotizing/non-caseating granulomata in lung and bone marrow confirming diagnosis of systemic sarcoidosis. Cultures from lung biopsy were negative for mycobacteria, fungal elements, or bacteria. The sarcoidosis staging was rated at stage 0 according to lung imaging, but with a high enough burden of disease to cause significant hypercalcemia. Please see Figure 3 for details of biopsy findings and bone marrow biopsy results. The patient started 20 mg of prednisone after diagnosis with improvement of hypercalcemia down to serum calcium of 10.2 mg/dL. She is receiving 3 times weekly hydration with IV normal saline to help prevent dehydration which helped decrease serum creatinine to 1.58 mg/dL. The patient was not on TNFi (tumor necrosis factor) to suggest secondary sarcoidosis. Her multiple sclerosis was stable as well. She was started on plaquenil for maintenance treatment of the diagnosed sarcoidosis. 

## Discussion 

We present this case to highlight several pathological findings that made this patient’s presentation noteworthy. The patient’s initial presentation suggested a non-parathyroid hormone-mediated cause of hypercalcemia, and workup did show elevated 1,25-dihydroxy vitamin D. While this suggested a granulomatous etiology, especially with persistent hypercalcemia and without any sign of other malignant driving process, confirming sarcoidosis by tissue diagnosis was more difficult. The lack of imaging findings was an obstacle to overcome, and the degree of hypercalcemia was unusual without any finding of hilar adenopathy. Ultimately the very elevated serum 1,25-dihydroxy vitamin D levels and angiotensin-converting enzyme levels helped increase the diagnostic suspicion to eventually obtain tissue and confirm sarcoidosis. 

Sarcoidosis presentations are variable, and imaging is often a crucial aid to diagnosis [3]. We reviewed several publications that stress that atypical diagnoses are often missed, especially since hypercalcemia frequency may be as low as 6% of sarcoidosis cases [12]. Cardiac sarcoidosis, ocular and neuro sarcoidosis may not have the classic presentation of hilar lymphadenopathy [3]. The variability of imaging findings should alert clinicians to the possibility of significant biochemical abnormalities from granulomas without findings of severe lymphadenopathy. We presented a case with hypercalcemia, severely increased 1,25-dihydroxy vitamin D, and angiotensin-converting enzyme levels that were elevated from diffuse granuloma without splenic or other systemic involvement. Despite the findings of diffuse granulomas, structural imaging was negative, and only PET scanning showed the right upper lobe findings and increased FDG uptake that suggested the diagnosis. The biochemical profile of elevated 1,25-dihydroxy vitamin D levels, hypercalcemia, and angiotensin-converting enzyme level should thus at least prompt functional imaging to rule out granulomatous disease. 

This case should spur clinicians’ awareness of the possibility of biochemical aberrations as the primary presentation of sarcoidosis, along with other silent presentations such as neuro-sarcoidosis, cardiac as well as ocular sarcoidosis. Ultimately, lung biopsy to confirm the presence of non-caseating granulomas is the gold standard, but some complications like post-biopsy pneumothorax may occur. The importance of obtaining tissue diagnosis in this case was to exclude infectious granulomatous causes such as fungal or mycobacterial infections that may have been serologically negative. Treatment with corticosteroids in such cases of occult infections could be hazardous. 

Besides sarcoidosis, mycobacterium tuberculosis, fungal disease resulting in granulomatosis, or other infectious presentations can present with findings similar to this case. *Mycobacterium avium intracellulare* (MAI, previously *Mycobacterium avium* complex) is one rare reported cause of hypercalcemia due to 1,25-dihydroxy vitamin D levels dysregulation due to granuloma formation [12]. Additionally, beryllosis and lymphoma are unusual causes of granulomatous disease [4, 13]. Foreign body reactions in sites other than the lungs have also produced granulomatous immune reactions in past. In one reported case, methylcrylate injections in the muscles produced refractory hypercalcemia with parathyroid suppression and granuloma formation similar to what we describe [14]. Moreover, anti-tumor necrosis factors can lead to secondary sarcoidosis. This leads to a clinical dilemma since this family is considered a therapeutic agent in sarcoidosis [15]. 

Finally it is important to also note that sarcoidosis is not the only autoimmune entity that has been known to result in granuloma formation and 1,25-dihydroxy vitamin D overproduction; as an example, a case of disseminated giant cell myositis resulted in a similar clinical phenotype [16]. Besides the lungs, possibly a clinician may have to consider other biopsy targets to identify the source(s) of granuloma formation when this source is not readily obvious. 

## Funding 

The authors have no funding to report in regards to this work. 

## Conflict of interest 

The authors declare that there is no conflict of interest regarding the publication of this paper.
